# Effect of SiN*_x_* diffusion barrier thickness on the structural properties and photocatalytic activity of TiO_2_ films obtained by sol–gel dip coating and reactive magnetron sputtering

**DOI:** 10.3762/bjnano.6.207

**Published:** 2015-10-16

**Authors:** Mohamed Nawfal Ghazzal, Eric Aubry, Nouari Chaoui, Didier Robert

**Affiliations:** 1 Laboratoire de physique des surfaces et interfaces, Université de Mons - UMONS, 20 Place du Parc, 7000 Mons, Belgium; 2 Institut Femto-ST (UMR 6174 CNRS), UFC, ENSMM, UTBM, 32 Avenue de l’Observatoire, 25044 Besançon Cedex, France; 3 LCP-A2MC, Institut Jean Barriol, Université de Lorraine, 1 Bd Arago, 57070 Metz, France,; 4 Laboratoire des Matériaux, Surfaces et Procédés pour la Catalyse, CNRS-UMR 7515, Antenne de Saint-Avold, Université de Lorraine, Rue Victor Demange, 57500 Saint-Avold, France

**Keywords:** diffusion barrier, photocatalysis, reactive sputtering, SiN*_x_*, sol–gel, titanium dioxide film, TiO_2_

## Abstract

We investigate the effect of the thickness of the silicon nitride (SiN*_x_*) diffusion barrier on the structural and photocatalytic efficiency of TiO_2_ films obtained with different processes. We show that the structural and photocatalytic efficiency of TiO_2_ films produced using soft chemistry (sol–gel) and physical methods (reactive sputtering) are affected differentially by the intercalating SiN*_x_* diffusion barrier. Increasing the thickness of the SiN*_x_* diffusion barrier induced a gradual decrease of the crystallite size of TiO_2_ films obtained by the sol–gel process. However, TiO_2_ obtained using the reactive sputtering method showed no dependence on the thickness of the SiN*_x_* barrier diffusion. The SiN*_x_* barrier diffusion showed a beneficial effect on the photocatalytic efficiency of TiO_2_ films regardless of the synthesis method used. The proposed mechanism leading to the improvement in the photocatalytic efficiency of the TiO_2_ films obtained by each process was discussed.

## Introduction

Titanium dioxide thin films in active phase (mostly anatase) have been widely studied due to their ability to produce strong oxidant species on the surface under UV light exposure [1] and to become super-hydrophilic [2]. One example of an application of this technology taking advantage of these combined properties is self-cleaning glass, which has transitioned from a promising technology to a global market product.

The contamination of titanium dioxide grown on soda lime glass (SLG) occurs during the calcination step and is due to the diffusion of alkali elements (especially sodium ions, Na^+^) [3–4]. Usually, TiO_2_ is amorphous when deposited at low temperature [5–6]. Heat treatment at a higher temperature (around 450 °C) is usually required in order to obtain the photoactive anatase phase. However, Na^+^ ions have a detrimental effect on the photocatalytic efficiency of TiO_2_ [3–4,7]. The poisoning effect of the Na^+^ ions on the photocatalytic activity occurs in different ways and depends on their concentration, for example: (a) Na^+^ ions increase the temperature of anatase formation and increase the particle size [4,7], (b) Na^+^ ions inhibit the formation of the anatase phase and act as a recombination center of photo-generated electron–hole pairs [3], and (c) Na^+^ ions induce the formation of brookite or sodium titanate (Na_2_O*_x_*TiO_2_), which is less photoactive than the anatase form [8]. In order to prevent this poisoning effect, various strategies have been reported including ion exchange via the formation of a thin proton-exchanged surface layer [3] or a post-treatment of the TiO_2_ films by hydrochloric acid [9], and the usage of a diffusion barrier intercalated between the substrate and the TiO_2_ film [6,10]. The most widely used diffusion barriers are SiO_2_ [10–11] and SiN*_x_* [6] layers. SiN*_x_* diffusion barriers show better efficiency than SiO_2_ with respect to inhibition of the diffusion of Na^+^ ions [6]. The sodium ion concentration (which diffuses at the film surface) is strongly related to the thickness of the SiN*_x_* layer [12]. Depending on the coating process, the thickness threshold required to prevent sodium ion diffusion and to guarantee a Na^+^-free TiO_2_ film is 100 nm [6] and 30 nm [12] for the reactive sputtered method and plasma-enhanced chemical vapor deposition, respectively.

In this paper, we studied the effect of the SiN*_x_* thickness on thin TiO_2_ layers coated by either a soft chemistry (sol–gel) or physical (reactive sputtering) method. In earlier work, we studied the concentration of Na^+^ ions as a function of the SiN*_x_* diffusion barrier thickness using extremely sensitive surface analysis techniques (X-ray photoelectron spectroscopy (XPS) and sputtered neutral mass spectrometry (SNMS)) [5–6,13]. Aubry et al. showed that the concentration of Na^+^ ions increases with a decrease of the SiN*_x_* diffusion barrier of thickness less than 150 nm [13]. Even though no surface analysis was performed in this study, we assume the Na^+^ ion concentration is reasonably correlated to the SiN*_x_* thickness (below 150 nm). In the present work, we report the unexpected effect of SiN*_x_* thickness (correlated to Na^+^ ion concentration) on the structural TiO_2_ films as a function of the preparation method. Finally, we discuss the effect of the SiN*_x_* thickness on the photocatalytic efficiency of the TiO_2_ films with the degradation of Orange II dye.

## Experimental

All of the reagents used in this work were of analytical grade and were used with no further purification and are as follows: titanium(IV) isopropoxide (TTIP) (Aldrich, 97%); ethanol absolute grade (99.9%); hydrochloric acid (37%) and Orange II (Sigma Chemical Co.).

### SiN*_x_* diffusion barrier

In order to prevent the diffusion of sodium ions from the SLG (which contains 14 wt % of Na_2_O), a SiN*_x_* layer was introduced between the TiO_2_ film and the SLG substrate. To determine the critical effect of the thickness of the SiN*_x_* diffusion barrier, the thickness of the barrier layer was varied by adjusting the reactive sputtering time using a custom in situ interferometry method described in detail elsewhere [14].

### Sol–gel dip coating of TiO_2_/SiN*_x_*/SLG

The TiO_2_ films prepared using the sol–gel process is described in detail elsewhere [7]. Briefly, titanium(IV) isopropoxide is used as a precursor to synthesize the TiO_2_ sol via an acid-catalyzed sol–gel process at room temperature by dissolving 10 mL of titanium(IV) isopropoxide in 50 mL of absolute ethanol under magnetic stirring. The hydrolysis of the precursor is catalyzed by adding 1.3 mL of HCl (Sigma-Aldrich, ACS reagent, 37%). The sol is stirred for 30 min and then a mixture of water (0.6 mL) and absolute ethanol (50 mL) are added dropwise, followed by magnetic agitation for an additional 2 h. The experiment was carried out under argon atmosphere.

The dip-coating process is used to grow the TiO_2_ films on SLG and on SLG coated with a SiN*_x_* diffusion barrier. The substrates were dipped into and pulled out of the sol at a speed of 11.5 cm·min^−1^. The films were dried in air at 70 °C for 5 min between each layer and the coating procedure was repeated as many times as necessary to obtain a 40 nm thickness. The films were dried overnight at 80 °C and then calcinated in air at 450 °C for 2 h with a heating rate of 5 °C·min^−1^.

### Reactive sputtering of TiO_2_/SiN*_x_*/SLG

The details of the sputtering procedure are described elsewhere [6]. TiO_2_ films were deposited on SLG and SiN*_x_*/SLG systems by direct current sputtering from a metallic Ti target in an Ar/O_2_ reactive gas mixture. The discharge current was regulated at 0.5 A and the resulting discharge voltage was 497 V. The total pressure was adjusted at 3.36 ± 0.07 Pa, while the Ar and O_2_ flow rates were fixed at 30 and 4 sccm, respectively. Special attention was paid to the controlled deposition of TiO_2_ films with a thickness in the range of 250–400 nm. The substrates were fixed on a rotating substrate holder that was parallel to the target surface. The SiN*_x_*/glass substrates were placed in the same position on the substrate holder (separated from the axis of the target by 50 mm at a fixed angle of 75°) to ensure reproducibility. Using cold deposition, the TiO_2_ coatings were calcinated at 450 °C for 2 h with a heating rate of 50 °C·min^−1^.

### Characterization

The coating morphology was investigated by scanning electron microscopy (SEM) using a Philips XL30 SFEG equipped with a field effect gun. The structural properties of the films were determined by X-ray diffraction (XRD) using Co Kα radiation at grazing incidence (0.05°). Silicon powder was dispersed on the coating surface in order to calibrate the diffractograms.

The photocatalytic activity of the films was evaluated by observing the photobleaching of Orange II (OII) dye with an initial concentration [OII] = 10 mg·L^−1^ over the course of 2 h as a pollutant model. The details of the procedure and the photoreactor used were reported elsewhere in detail [7]. The photoreactor was placed in a solar box (Atlas, Suntest CPS+) equipped with a Xe lamp as a light source (300 nm < λ < 800 nm) to simulate natural UV–vis radiation. An incident power density of 23.3 W·m^−2^ was measured by a power meter at the sample location. Before each experiment, a blank reference with UV–vis light illumination of an SLG substrate without a TiO_2_ coating was performed. After 2 h of exposure, the observed decrease in the peak at 485.5 nm was less than 0.02% of the initial absorbance.

The bleaching of the dye was thus quantitatively evaluated by recording the “real time” evolution of the maximum absorbance value of the OII, at 485.5 nm, at neutral pH 7.2, using a quartz circulating cell placed in a UV–vis spectrometer. The photocatalytic activity of the films was quantitatively evaluated by comparing the bleaching reaction rates. The degradation rate can be described by a pseudo-one-order model when the dye concentration is low as, ln *C*_0_/*C* = *k*_a_*t*, where *C*_0_ is the initial dye concentration (mg·L^−1^).

## Results and Discussion

### TiO_2_ films morphology

**Figure 1:**
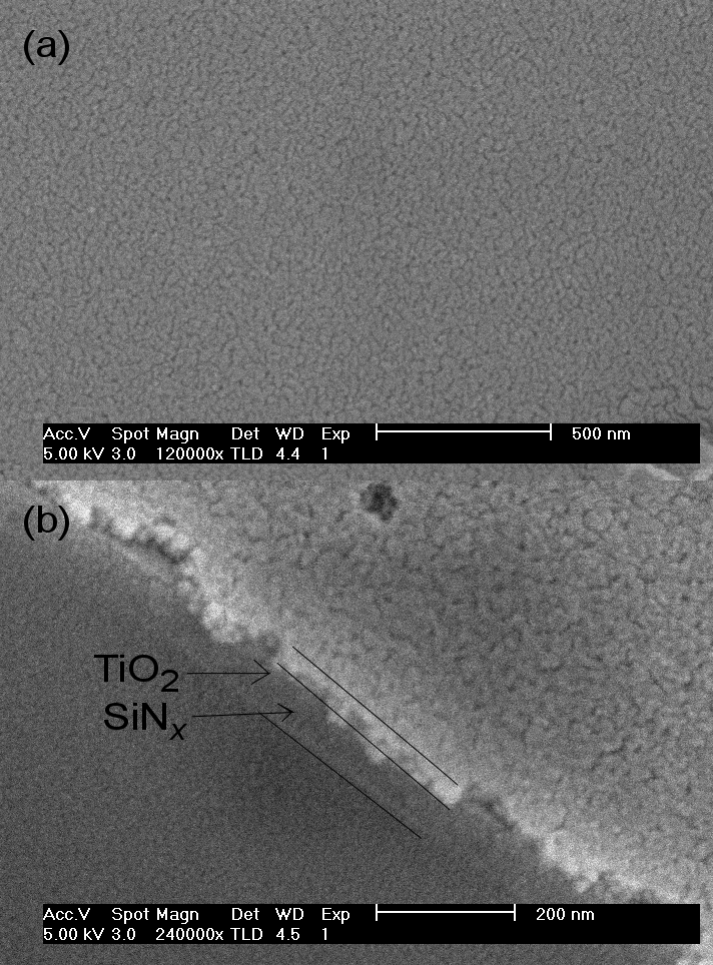
SEM images of the (a) top surface and (b) cross section of the sol–gel TiO_2_ films grown on 75 nm thick SiN*_x_*/SLG after annealing at 450 °C.

Figure 1 shows SEM images highlighting the TiO_2_ film morphology resulting from the sol–gel process and calcination at 450 °C for an increasing SiN*_x_* barrier thickness. The surface of the film resulting from the sol−gel method was uniform. The average grain size was about 30−50 nm when a TiO_2_ film was directly grown on the SLG. The grain size appeared to be reduced and the thickness of the SiN*_x_* barrier decreased. The evolution of the grain size of TiO_2_ nanoparticles is also confirmed by the results extracted from the XRD patterns (Figure 5a). The film thickness, estimated from the cross section, indicates that the films are about 40 nm thick (Figure 1b).

**Figure 2:**
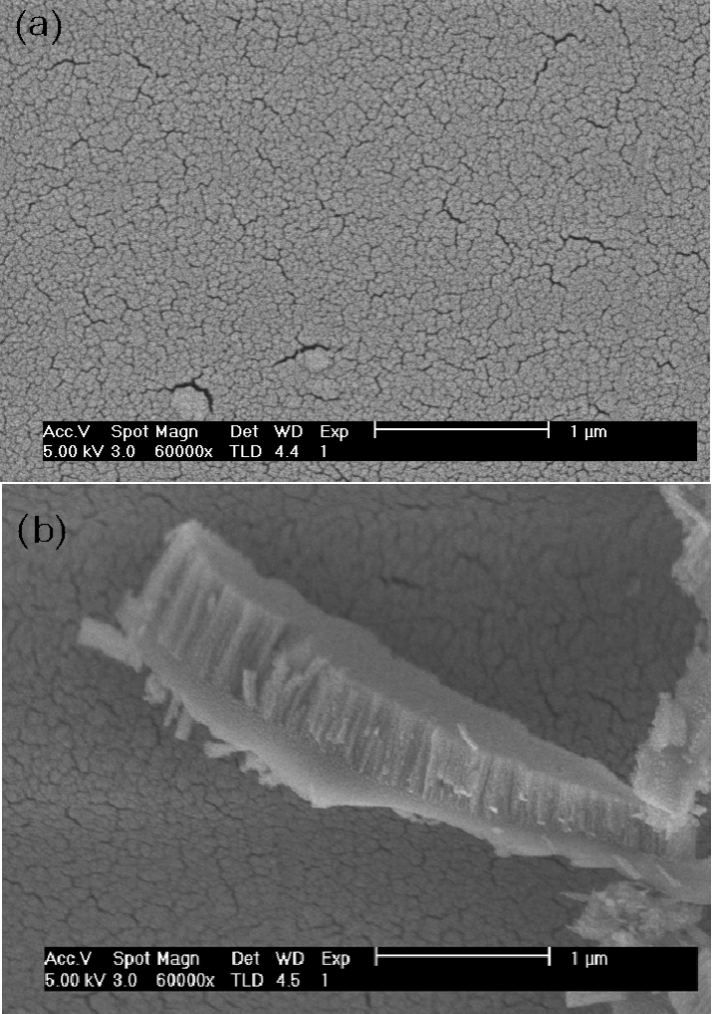
SEM images of (a) the top surface and (b) the cross-section of the magnetron sputtered TiO_2_ films grown on SiN*_x_*/SLG after annealing at 450 °C.

The surface morphology of the annealed TiO_2_ coatings synthesized on SiN*_x_*/SLG substrates by reactive sputtering is presented in Figure 2. The film surface does not show a major difference when the thickness of SiN*_x_* barrier increases. The coatings treated at 450 °C show small cracks on the surface. The cracks are the same regardless of barrier thickness. This could be a consequence of the accumulation of intrinsic tensile stress induced by the crystallization of TiO_2_ [7]. As deposited on SiN*_x_*/SLG following the described procedure, the TiO_2_ films are amorphous and an annealing step at high temperature is needed to obtain the photoactive phase (anatase). The growth of TiO_2_ in its anatase form induces an internal tensile stress, which creates cracks in the films. The average grain size of TiO_2_ is estimated to be 20–40 nm, according to the SEM images. The average grain size is not affected by the variation of the SiN*_x_* barrier thickness. From the fragmented cross-sections (Figure 2b), the film morphology appeared to be composed of distinguishable columns separated by boundaries, which corresponds to an intracolumnar porosity. Operating at pressures higher than the dense-to-columnar transition pressure (used in our study) enable such a structure to be obtained. The resulting films exhibited a high specific surface area.

### TiO_2_ film structure

**Figure 3:**
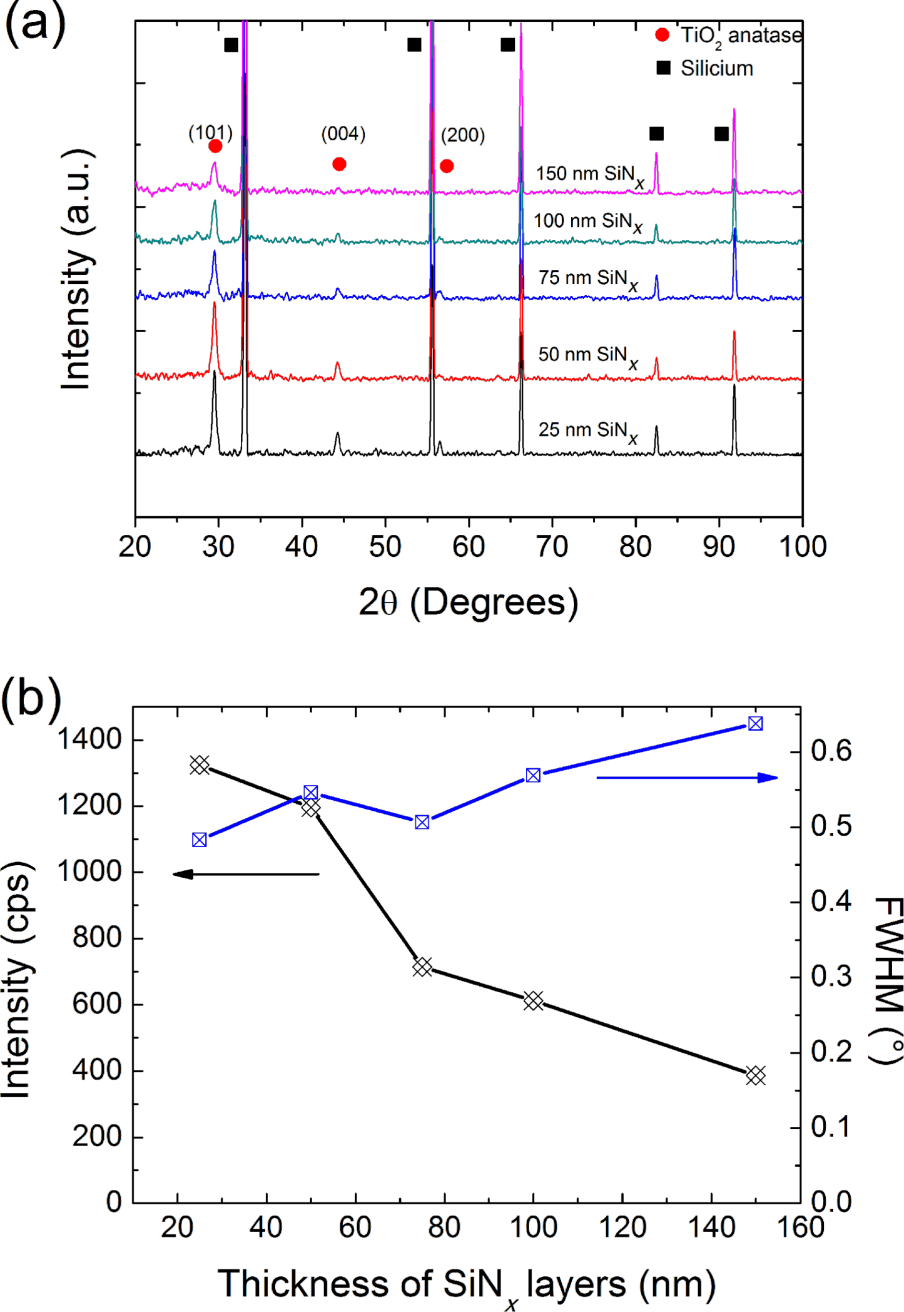
(a) XRD patterns and (b) FWHM and the intensity of the (101) plane of the sol–gel TiO_2_ coatings as a function of the SiN*_x_* layer thickness.

**Figure 4:**
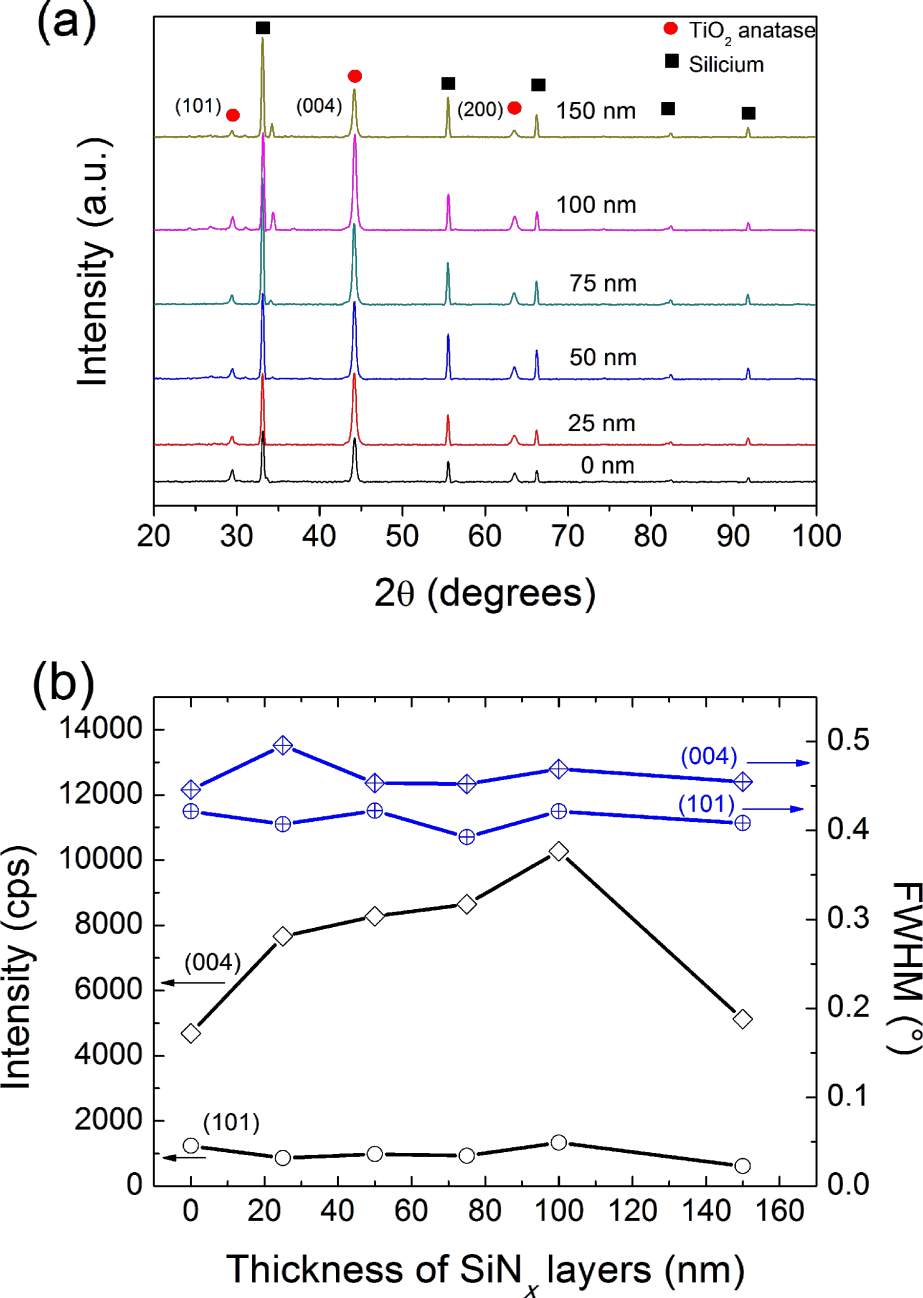
(a) XRD patterns and (b) FWHM of the (101) and (004) planes and their intensity of the TiO_2_ coatings as a function of the thickness of the SiN*_x_*/SLG films.

The X-ray diffraction (XRD) patterns of the TiO_2_ films grown on SiN*_x_*/SLG using different methods and heat treated at 450 °C are presented in Figure 3 and Figure 4. It is worth noting that the films, as deposited on SLG or on SiN*_x_*/SLG systems, are amorphous regardless of the deposition method. After the calcination step, TiO_2_ grown on SLG is crystallized in the anatase structure regardless of the method and the SiN*_x_* diffusion barrier does not affect the crystallization of the films. This result suggested that the SLG substrate does not affect the crystallization step of the amorphous TiO_2_ films, which is in contrast with previous reports. Novota et al. found that TiO_2_ films deposited on SLG exhibited a brookite dominant crystalline phase [10]. Koo et al. reported that a SiN*_x_* diffusion barrier is necessary to crystallize VO_2_ films [12]. The XRD patterns reveal a preferential [101] orientation for the sol–gel TiO_2_ films, whereas the reactive sputtered TiO_2_ films show a [001] preferential orientation. In order to understand the effect of the diffusion barrier thickness on the structure of the TiO_2_ films, the full width at half maximum (FWHM) of the preferential reflection is presented in Figure 3b and Figure 4b.

Two distinct behaviors are observed depending on the coating method. While the FWHM of the [101] diffraction patterns of the TiO_2_ made by the sol–gel method decreased with decreasing diffusion barrier thickness as compared to that measured for the [101] and [004] diffraction plans in the case of TiO_2_ films made by reactive sputtering, which do not show a significant change. The intensity of the peak diffraction decreased for the sol–gel coating, whereas it remained unchanged for the film made by the reactive sputtering method. According to Scherrer's equation [15], the crystallite size was estimated for the TiO_2_ sol–gel (SG-TiO_2_) coating and the magnetron sputtered TiO_2_ (MS-TiO_2_) grown on the glass and on SiN*_x_*/glass was compared by measuring the FWHM of the (101) diffraction peaks. It was found that for MS-TiO_2_ samples, the presence of the diffusion barrier does not affect the crystallites size, which was about 24–30 nm in both cases. This result is in agreement with our previous study but for a much thicker SiN*_x_* diffusion barrier [6]. This suggests that the crystallite size of the TiO_2_ films obtained by magnetron sputtering does not depend on the thickness of the SiN*_x_*. Furthermore, no difference has been observed between the crystallite size of the MS-TiO_2_ films grown on SLG or on SiN*_x_*/SLG, which suggested that the SiN*_x_* layer does not affect the crystallite growth of the TiO_2_ during the heat treatment. However, the crystallite size of SG-TiO_2_ films decreases gradually as the thickness of the SiN*_x_* diffusion barrier increases. TiO_2_ thin films grown on SLG were obtained and discussed in our previous study [7]. The TiO_2_/SLG sample presented a granular structure with a grain size of ≈30–50 nm. The crystallite started with a size around 30–50 nm when SG-TiO_2_ was grown on SLG, which decreased to approximately 18 nm. Similar results were observed for TiO_2_ film obtained by sol–gel methods on various substrates. Nam et al. reported that the Na^+^ ions in the TiO_2_ films induce an increase in their crystallite size [4]. The diffusion of the ions at the grain boundary during the heat treatment is in competition with the nucleation/growth of the anatase crystallite, which induces an increase in the temperature required for nucleation, while the activation energy of the grain growth decreases [16]. The efficiency of the SiN*_x_* diffusion barrier to inhibit sodium diffusion from the SLG must be related to its thickness. It is well known that the SLG contains 14 wt % of Na_2_O, and the calcination at high temperature of the TiO_2_ films induces the diffusion of 8% of the Na^+^ ions from the substrate to the TiO_2_ surface [17]. Aubry and coworkers [6] reported that the SiN*_x_* diffusion barrier with a 100 nm thickness is sufficient to hinder the alkali diffusion (only 2% of sodium ions were detected by SNMS in the TiO_2_ film surface), and increasing the thickness up to 1,000 nm does not further reduce the concentration of Na^+^ ions. Koo et al. [12] showed that the SiN*_x_* diffusion barrier contributes to the formation of the VO_2_ crystalline phase. From XPS results, it was concluded that the amount of sodium ions diffused to the top VO_2_ layer decreases gradually as the thickness decreases from 100 nm to 10 nm. Increasing the SiN*_x_* diffusion barrier thickness indirectly induces the decrease in the crystallite size of the SG-TiO_2_ film. A similar behavior has been observed for TiO_2_ films grown on various types of glasses containing different concentrations of sodium in their composition [4,7].

### Photocatalytic efficiency of SG-TiO_2_ and MS-TiO_2_ films as a function of SiN*_x_* diffusion barrier thickness

**Figure 5:**
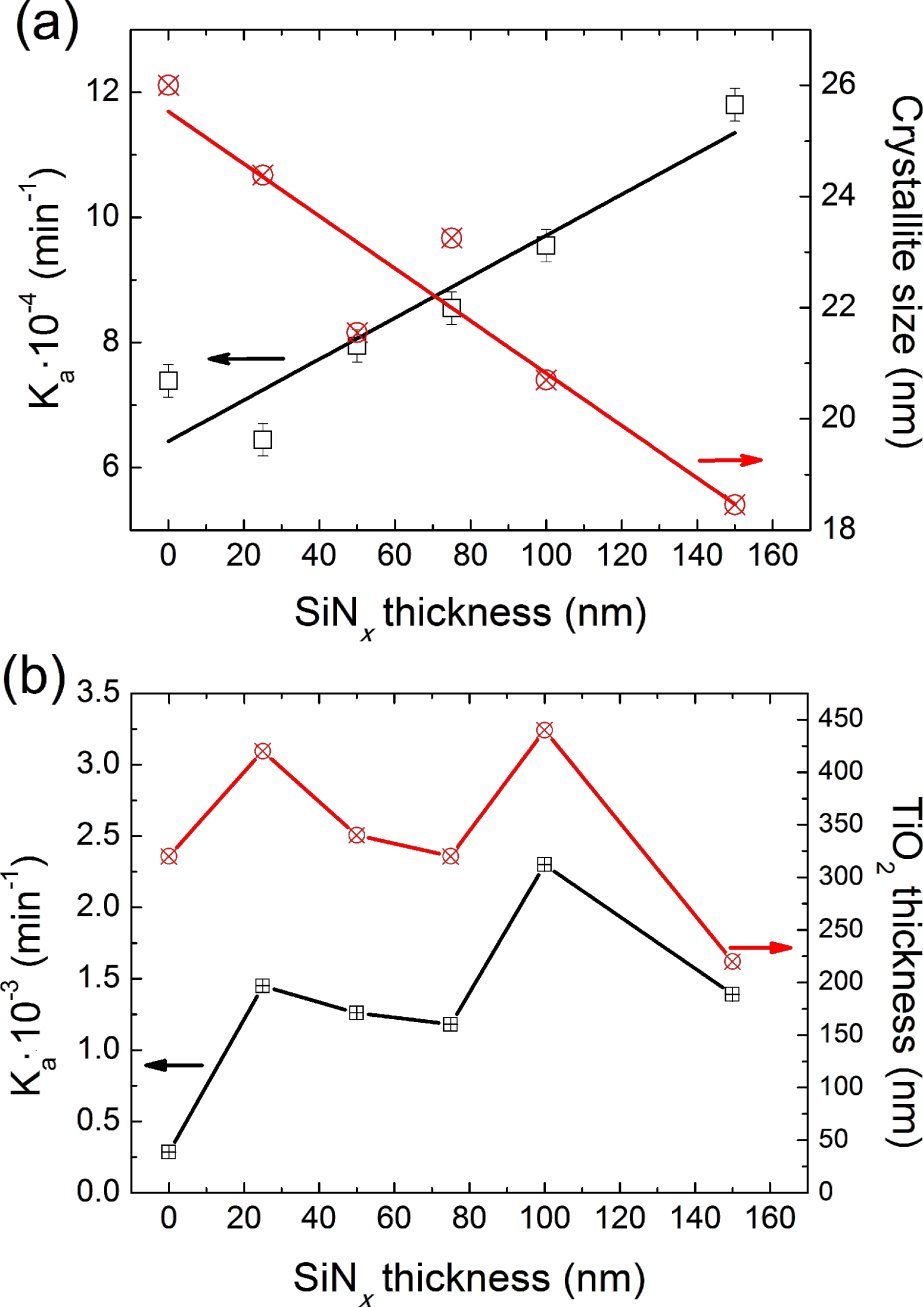
Evolution of the kinetics of the degradation rate of OII. The crystallite size and TiO_2_ thickness of (a) a SG-TiO_2_ film and (b) the magnetron sputtered TiO_2_ film as a function of SiN*_x_* thickness.

The photocatalytic activity of the films was evaluated by following the photobleaching reaction rate of OII dye as a function of the irradiation time (Figure 5). According to our results, the reaction rate follows a Langmuir–Hinshelwood kinetic, which can be described by a pseudo-first-order model since the dye concentration is low. No significant change was observed for the initial absorbance (concentration) of OII in the presence of an uncoated SLG under UV–vis illumination or in the dark in the presence of the TiO_2_ photocatalyst. Thus, the OII was not photobleached by photolysis nor was it adsorbed at the surface of the photocatalyst, which suggests a neglected effect of the specific surface area of the film on the photocatalytic efficiency. The photobleaching of the OII dye in the presence of the catalyst was performed at neutral pH 7.2 during 2 h of UV–vis illumination. At neutral pH, the bleaching of OII was caused by photo-oxidation via formation of radicals and/or a UV-induced modification of the surface, leading to the adsorption/bleaching of the azo molecule [18]. This means that even the dye molecule OII could absorb visible light to produce the excited singlet and/or triplet state of the OII molecule [19], the OII may be able to sensitize the TiO_2_ photocatalyst. However, the degradation of the OII dye via the sensitization mechanism has only a minor contribution to the overall degradation rate [7].

The results are depicted as the apparent constant rate of the OII degradation as a function of the illumination time. A different behavior for SG-TiO_2_ and MS-TiO_2_ films was observed. For the films made by the sol–gel method, the degradation rate of the OII dye increases with increasing thickness of the SiN*_x_* diffusion barrier. This suggests that the kinetic of the degradation of the OII dye is inversely proportional to the crystallite size of TiO_2_. Reducing the crystallite size of the SG-TiO_2_ (from 30–50 to 18 nm) by increasing the SiN*_x_* diffusion barrier thickness affects the interfacial electron transfer rate [20]. The photo-generated electron–hole pair produced in the bulk of the photocatalyst during the illumination diffuses faster to reach the surface, since the distance traveled is reduced by the smaller crystallite, and redox reactions with species present on the surface takes place. This leads to the rapid formation of radical species (O_2_**^•−^**, HO_2_**^•^**, HO**^•^**), followed by a rapid degradation of the OII dye. For the MS-TiO_2_ system, once the SiN*_x_* diffusion barrier has been intercalated between the TiO_2_ film and the SLG substrate, the degradation kinetic rate of OII shows a sudden improvement. The degradation rate of OII becomes five times greater than that of the TiO_2_ film grown solely on SLG with a SiN*_x_* layer of about 25 nm, and then the degradation rate of the OII dye becomes independent of the diffusion barrier thickness. In this case, the crystallite size of the TiO_2_ photocatalyst is not responsible for the change in photocatalytic efficiency. This result suggests that SiN*_x_* inhibits the diffusion of Na^+^ ions into the films during the calcination step, which is actually a well-documented fact [4,7,21]. Tada and Tanaka [21] attributed the clear difference observed in the photocatalytic activity between sol–gel TiO_2_ films grown on quartz and SLG to the presence of Na^+^ ions acting as recombination centers for the photocarriers. Moreover, Koo et al. [12] show a clear correlation between the SiN*_x_* diffusion barrier thickness and the Na^+^ ion concentration on the surface of the films during the calcination step. This suggests that a 25 nm SiN*_x_* diffusion barrier is sufficient to inhibit sodium diffusion. Consequently, more electron–hole pairs are photo-generated at the TiO_2_ surface, leading to an increase in the radical species responsible for the degradation of OII. For an equivalent TiO_2_ thickness, the degradation rate of OII is relatively similar.

## Conclusion

We investigated the structural and photocatalytic properties of titanium dioxide films obtained by low temperature sol–gel and reactive sputtering processes for SiN*_x_* diffusion barriers of different thicknesses. The structural properties of the TiO_2_ films was affected by the process used for the production of the films. The preferential orientation of the anatase phase obtained for the samples produced from the sol–gel process is the (001) plane, whereas those from the reactive magnetron sputtering process show a preferential (004) orientation plane. Regardless of the process used to synthesize the TiO_2_ films, the intercalating SiN*_x_* diffusion barrier between the photocatalyst and the soda lime glass showed a beneficial effect on the photocatalytic efficiency. An increased SiN*_x_* diffusion barrier thickness resulted in a decrease in the crystallite size of the TiO_2_ film when produced by the sol–gel method, and consequently, the photocatalytic degradation of the OII dye was improved. However, when the reactive sputtering method was used, the thickness of the diffusion barrier had no effect on the structural properties of the TiO_2_ films.
